# Detecting Parkinson Disease Using a Web-Based Speech Task: Observational Study

**DOI:** 10.2196/26305

**Published:** 2021-10-19

**Authors:** Wasifur Rahman, Sangwu Lee, Md Saiful Islam, Victor Nikhil Antony, Harshil Ratnu, Mohammad Rafayet Ali, Abdullah Al Mamun, Ellen Wagner, Stella Jensen-Roberts, Emma Waddell, Taylor Myers, Meghan Pawlik, Julia Soto, Madeleine Coffey, Aayush Sarkar, Ruth Schneider, Christopher Tarolli, Karlo Lizarraga, Jamie Adams, Max A Little, E Ray Dorsey, Ehsan Hoque

**Affiliations:** 1 Department of Computer Science University of Rochester Rochester, NY United States; 2 Center for Health and Technology University of Rochester Medical Center Rochester, NY United States; 3 Department of Neurology University of Rochester Medical Center Rochester, NY United States; 4 School of Computer Science University of Birmingham Birmingham United Kingdom; 5 MIT Media Lab Massachusetts Institute of Technology Cambridge, MA United States

**Keywords:** Parkinson’s disease, speech analysis, improving access and equity in health care, mobile phone

## Abstract

**Background:**

Access to neurological care for Parkinson disease (PD) is a rare privilege for millions of people worldwide, especially in resource-limited countries. In 2013, there were just 1200 neurologists in India for a population of 1.3 billion people; in Africa, the average population per neurologist exceeds 3.3 million people. In contrast, 60,000 people receive a diagnosis of PD every year in the United States alone, and similar patterns of rising PD cases—fueled mostly by environmental pollution and an aging population—can be seen worldwide. The current projection of more than 12 million patients with PD worldwide by 2040 is only part of the picture given that more than 20% of patients with PD remain undiagnosed. Timely diagnosis and frequent assessment are key to ensure timely and appropriate medical intervention, thus improving the quality of life of patients with PD.

**Objective:**

In this paper, we propose a web-based framework that can help anyone anywhere around the world record a short speech task and analyze the recorded data to screen for PD.

**Methods:**

We collected data from 726 unique participants (PD: 262/726, 36.1% were women; non-PD: 464/726, 63.9% were women; average age 61 years) from all over the United States and beyond. A small portion of the data (approximately 54/726, 7.4%) was collected in a laboratory setting to compare the performance of the models trained with noisy home environment data against high-quality laboratory-environment data. The participants were instructed to utter a popular pangram containing all the letters in the English alphabet, “the quick brown fox jumps over the lazy dog.” We extracted both standard acoustic features (mel-frequency cepstral coefficients and jitter and shimmer variants) and deep learning–based embedding features from the speech data. Using these features, we trained several machine learning algorithms. We also applied model interpretation techniques such as Shapley additive explanations to ascertain the importance of each feature in determining the model’s output.

**Results:**

We achieved an area under the curve of 0.753 for determining the presence of self-reported PD by modeling the standard acoustic features through the XGBoost—a gradient-boosted decision tree model. Further analysis revealed that the widely used mel-frequency cepstral coefficient features and a subset of previously validated dysphonia features designed for detecting PD from a verbal phonation task (pronouncing “ahh”) influence the model’s decision the most.

**Conclusions:**

Our model performed equally well on data collected in a controlled laboratory environment and *in the wild* across different gender and age groups. Using this tool, we can collect data from almost anyone anywhere with an audio-enabled device and help the participants screen for PD remotely, contributing to equity and access in neurological care.

## Introduction

Parkinson disease (PD) is the fastest-growing neurological disease worldwide. Unfortunately, an estimated 20% of patients with PD remain undiagnosed. This can be largely attributed to the shortage of neurologists worldwide [1,2] and limited access to health care. Early diagnosis and continuous monitoring, which allows medication dosage adjustment, are key to managing the symptoms of this incurable disease. The current standard of diagnosis requires in-person clinic visits where an expert assesses the disease symptoms while observing the patients as they perform tasks from the Movement Disorder Society-Unified Parkinson’s Disease Rating Scale (MDS-UPDRS) [3]. The MDS-UPDRS includes 24 motor-related tasks to assess speech, facial expression, limb movements, walking, memory, and cognitive abilities. Although many studies have shown success by analyzing hand movements [4], limb movement patterns [5], and facial expressions [6], speech is especially important because approximately 90% of patients with PD exhibit vocal impairment [7,8], which is often one of the earliest indicators of PD [9].

For speech analysis, researchers have looked into phonations (pronouncing “ahhh”) from audio recordings [10] to quantify rhythm, stress, and intonation [11]. Little et al [12] introduced pitch period entropy (PPE) as a measure of dysphonia to distinguish healthy people from people with PD with 91% accuracy. Later, Tsanas [13] expanded on this by calculating 132 dysphonia measures to classify PD versus control with almost 99% accuracy. In addition, Peker et al [14] used a novel feature selection technique with a complex-valued artificial neural network. Rueda and Krishnan [15] identified a set of mel-frequency cepstral coefficients (MFCCs) and intrinsic mode functions to represent the characteristics of PD. In the domain of analyzing real-life audio data, Wroge et al [16] analyzed verbal phonation data collected from smartphones; Vaiciukynas et al [17] used a convolutional neural network to detect PD from speech; Vásquez-Correa et al [18] collected speech samples with a handheld device in uncontrolled noise conditions; and Dubey et al [19] designed a smartwatch-based voice and speech monitoring system for people with PD receiving speech therapies from speech-language pathologists. Although the current state-of-the-art method has shown promising results, it has limitations such as small sample size [12,19,20], oversampling from the same participants [14], noise-controlled data collection [12,15], and age discrepancy between PD and control [21].

In this paper, we present our analysis of 726 audio recordings of speech from 36.1% (262/726) individuals with PD and 63.9% (464/726) without PD. The speech recordings were collected using a web-based tool called *Parkinson’s Analysis with Remote Kinetic-tasks* (PARK) [22]. The PARK tool instructed the participants to utter a popular pangram containing all the letters in the English alphabet, “The quick brown fox jumps over the lazy dog,” and recorded it. This allowed us to rapidly collect a data set that is more likely to contain real-world variability associated with geographical boundaries, socioeconomic status, age groups, and a wide variety of heterogeneous recording devices. The findings in this study build on this unique real-world data set; thus, we believe it could potentially be generalized for real-world deployments.

Collecting audio data from individuals often requires in-person visits to the clinic, limiting the number of data points and the diversity within the data. Recent advancements have allowed the collection of tremor data from wearable sensors [23] and sleep data from radio frequency signals [24]. The existing work with speech and audio analysis uses sophisticated equipment for collecting data that are often noise-free [12,25] and do not contain real-world variability. As a significant portion of the population has access to a mobile device with recording capability (eg, 81% of Americans own a smartphone) [26], we opted to use a framework that allows participants to record data from their homes. From the recorded audio files, we extracted acoustic features including MFCCs, which represent the short-term power spectrum of a sound, jitter or shimmer variants (representing pathological voice quality), pitch-related features, spectral power, and dysphonia-related features designed to capture PD-induced vocal impairment [12].

In addition, we extracted features from a deep-learning–based encoder—problem agnostic speech encoder (PASE) [27], which represents the information contained in a raw audio instance through a list of encoded vectors. These features are modeled with four different machine learning models—support vector machine (SVM), random forest, LightGBM, and XGBoost—to classify individuals with and without PD.

**Figure 1:**
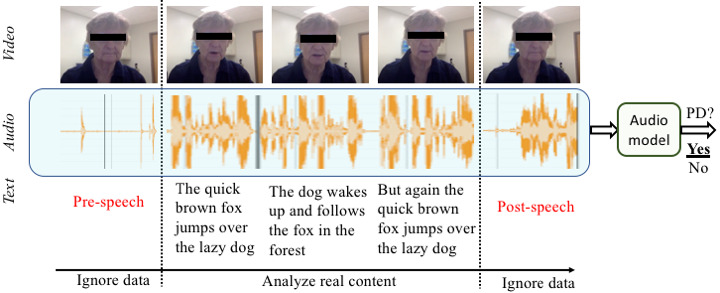
An outline of our approach for solving the speech task of uttering “The quick brown fox...”.

Figure 1 provides an outline of the data analysis system. Our contributions can be summarized as follows:

- We report findings from one of the largest data sets with real-world variability, containing 726 unique participants mostly from their homes.
- We analyzed the audio features of speech to predict PD versus non-PD with an area under the curve (AUC) score of 0.753.
- We provide evidence that our model prioritizes MFCC features and a subset of dysphonia features [12,28], which is consistent with previous literature.
- Our model performs consistently well when tested on gender- and age-stratified data collected in a controlled laboratory environment and in the wild.

## Methods

### Recruitment and Data Collection

**Figure 2:**
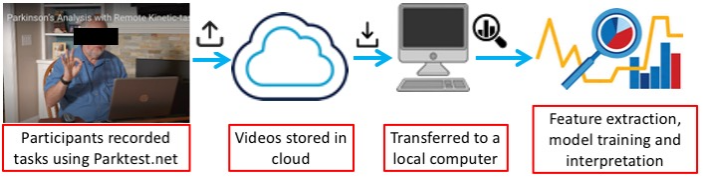
An overview of our data collection, storage, and analysis pipeline.

**Figure 3:**
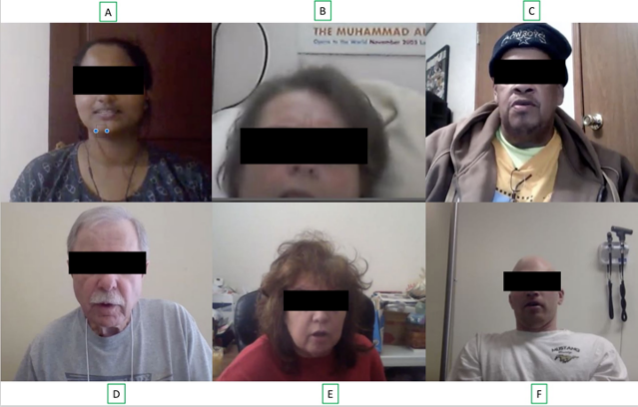
Some screenshots of our subjects while providing the data. All the subjects except B provided data without any supervision. B, D, E, and F have been diagnosed with Parkinson disease. Electronic informed consent was taken from the participants to use their photos for publication.

**Figure 4:**
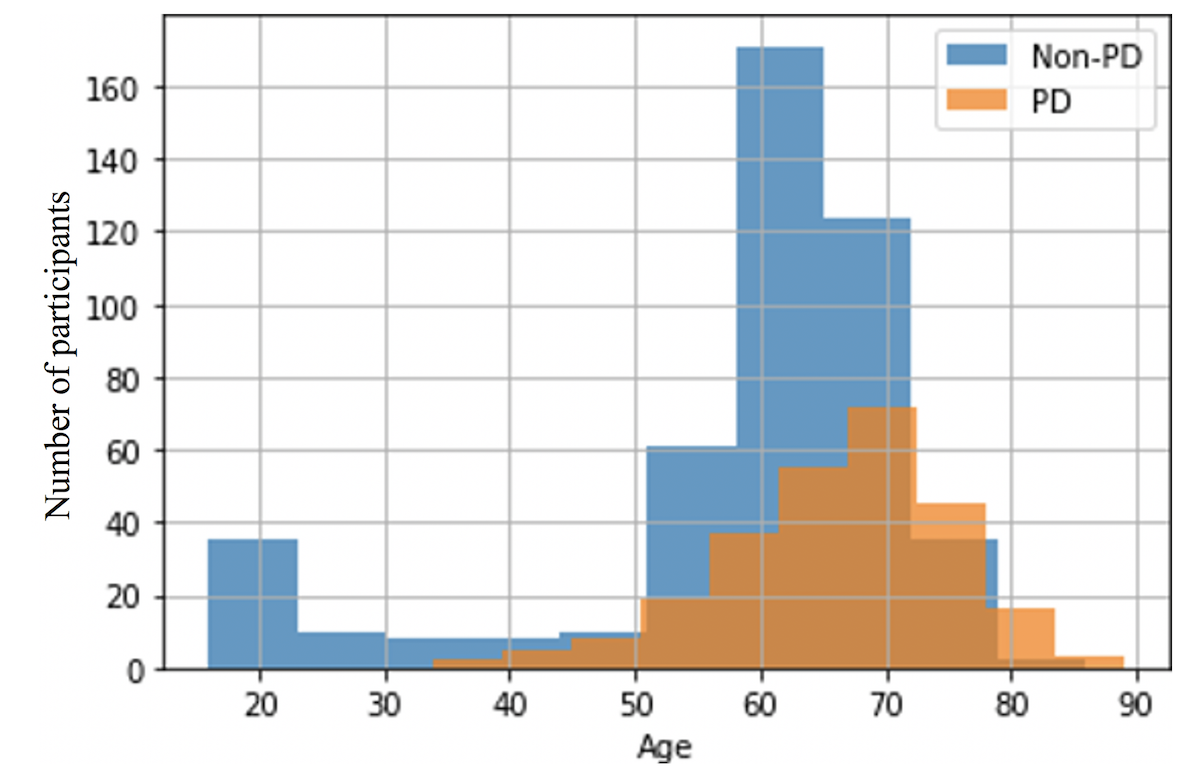
A bar plot showing the age distribution of participants with PD and those without PD in our data set. PD: Parkinson disease.

We collected data from 726 *unique* participants uttering the sentences, “The quick brown fox jumps over the lazy dog. The dog wakes up and follows the fox into the forest, but again the quick brown fox jumps over the lazy dog,” using PARK [29] website. Figure 2 provides a brief overview of the data collection, storage, transfer, and analysis mechanisms. The users are recorded via a webcam and a microphone connected to the PC or laptop, and the recordings are uploaded to a server. Figure 3 shows images of some of the study participants, and Figure 4 shows the age distribution of the participants. The number of participants without PD is 1.8 times the number of participants with PD. Most of the participants with PD are concentrated in the age range of 40 to 80 years. However, participants without PD outnumber those with PD in the age group 20 to 40 years. Similarly, participants with PD significantly outnumber those without PD in the age group of 80 to 90 years. Table 1 provides the demographic information of the study participants.

**Table 1:** Demographic composition of our data set (N=726).

Characteristics		Participants with PD^a^	Participants without PD
Total, n (%)		262 (36.1)	464 (63.9)
**Gender, n (%)**			
	Female	101 (38.5)	300 (64.6)
	Male	161 (61.4)	164 (22.5)
Age (years), mean (SD)		65*.*92 (9*.*2)	57*.*98 (14*.*2)
**Country, n (%)**			
	United States	199 (75.9)	419 (90.3)
	Other	63 (24)	45 (9.7)
Years since diagnosed, mean (SD)		7*.*88 (5*.*41)	N/A^b^

^a^PD: Parkinson disease.

^b^N/A: not applicable.

Among our 726 unique participants, 262 (36.1%) had received the diagnosis of PD, whereas the others were participants without PD. We obtained contact information of patients with PD from local clinics and various PD support groups. Participants without PD were recruited from Amazon Mechanical Turk. During data collection, informed consent of all participants was obtained in accordance with the protocol agreed upon between the researchers and the institutional review board of the University of Rochester. Among the 726 unique participants, only 54 (7.4%) provided data in the laboratory under the guidance of a study coordinator using the PARK tool; the rest of the 672 (92.6%) participants used the PARK system from their homes to provide data. Having participants perform the tasks at home and in the laboratory allowed us to compare the results across both conditions. No participant appeared in either set, and *all* of our participants used the identical PARK protocol.

The gender distribution in the data set was skewed, especially for female participants. Among all participants, 55.2% (401/726) were women, and 44.8% (325/726) were men. However, among participants with PD, only 38.5% (101/262) were women, and for participants without PD, 64.6% (300/464) were women. Most of the participants with PD were in the age range of 40 to 80 years, but most of the younger (20-40 years) and older (80-90 years) participants were from the group without PD and the group with PD, respectively.

As our data were collected through a web-based framework, we do not have the MDS-UPDRS scores for our participants because collecting them requires additional input from doctors. Among all the participants in our PD group, only 3 participants replied in affirmative that they had taken medication 2 hours before taking the test; others replied in the negative. Therefore, we assumed that medication effects were negligible for the purposes of this study.

### Data Preprocessing

During data collection, participants often took some additional time to start to utter the task sentences and stop the recording once the sentences were uttered. Hence, we had a substantial amount of noisy and irrelevant data at the beginning and end of most data instances (Figure 1). To remove irrelevant data, we used the Penn Phonetics Lab Forced Aligner Toolkit (P2FA) [30]. Given an audio file and transcript, it attempts to predict the time boundaries in which each of the words in the transcript was pronounced. P2FA applies a combination of hidden Markov models [31] to predict the most likely sequence of phonemes for a given audio and Gaussian mixture models to combine those phonemes into words and obtain their time boundary using a predefined dictionary [32]. From the output of this system, we can obtain the starting time of the first word recognized by the P2FA and the ending time of the last word recognized by the P2FA. We used the audio segments between them for further analysis.

### Acoustic Features Extraction

We extracted features by combining outputs of multiple sources: Praat features [33] obtained through the Parselmouth Python interface [34] and the previously used dysphonia features relevant for PD analysis [12,35,36]. After calculating all the features, we constructed a correlation matrix of the feature values to calculate the degree of correlation between them. Then, we iterated over each pair of features in an unordered fashion, and if the correlation coefficient between them was above 0.9, we dropped one of those features from further analysis [37]. Table 2 contains a short overview of the features used in our analysis; the feature names in italicized text are those used for building the models. We provide a more comprehensive description of the features in Multimedia Appendix 1 [12,14,36,38]. Some of our definitions were adapted from the official Praat documentation [33].

**Table 2:** Names of all the features, code source used for collecting them, and a short description^a^.

Feature			Code source		Short description
**Pitch**					
	*MedianPitch* ^b^	Little et al [36]		Median principal frequency	
	*MeanPitch*	Boersma and Weenink [33]		Mean principal frequency	
	*StdDevPitch*	Little et al [36]		SD in principal frequency	
**Jitter**					
	*MeanJitter*	Little et al [36]		Perturbation in principal frequency (mean variation)	
	*MedianJitter*	Little et al [36]		Perturbation in principal frequency (median variation)	
	LocalJitter	Boersma and Weenink [33]		Average absolute difference between consecutive periods, divided by the average period	
	*LocalAbsoluteJitter*	Boersma and Weenink [33]		Average absolute difference between consecutive periods measured in seconds	
	RapJitter	Boersma and Weenink [33]		Average absolute difference between a period and the average of it and its two neighbors	
	Ppq5Jitter	Boersma and Weenink [33]		5-point period perturbation quotient	
	*DdpJitter*	Boersma and Weenink [33]		Difference of differences of periods of principal frequency	
**Shimmer**					
	MeanShimmer	Little et al [36]		Amplitude perturbation (using mean)	
	*MedianShimmer*	Little et al [36]		Amplitude perturbation (using median)	
	LocalShimmer	Boersma and Weenink [33]		Average absolute difference between amplitudes of consecutive period	
	LocaldbShimmer	Boersma and Weenink [33]		Average absolute base-10 logarithm of the difference between amplitudes of consecutive period	
	Apq3Shimmer	Boersma and Weenink [33]		3-point amplitude perturbation quotient	
	Apq5Shimmer	Boersma and Weenink [33]		5-point amplitude perturbation quotient	
	*Apq11Shimmer*	Boersma and Weenink [33]		11-point amplitude perturbation quotient	
	*DdaShimmer*	Boersma and Weenink [33]		Shimmer calculated by difference in differences of amplitude	
**MFCC^c^**					
	*MeanMFCC (0-12)*	Little et al [36]		13 features of mean MFCC	
	*VariationMFCC (0-12)*	Little et al [36]		13 features of mean variation of MFCC	
	*RelBandPower (0-3)*	Tsanas et al [39]		4 features capturing relative band power in 4 spectrum ranges	
*HNR* ^d^			Boersma and Weenink [33]		Signal-to-noise ratio
*RPDE* ^e^			Little et al [36]		Pitch estimation uncertainty
*DFA* ^f^			Little et al [36]		Measure of stochastic self-similarity in turbulent noise
*PPE* ^g^			Little et al [36]		Measure of inability of maintaining constant pitch

^a^We collected the features using the code or methodology described in the corresponding code-source entry.

^b^The correlated features were removed, and features in italicized text were used to build the models. Feature names are preceded by the loosely defined umbrella category they belong to.

^c^MFCC: mel-frequency cepstral coefficient.

^d^HNR: harmonic-to-noise ratio.

^e^RPDE: recurrence period density entropy.

^f^DFA: detrended fluctuation analysis.

^g^PPE: pitch period entropy.

### Embedding Features Extraction

We extracted deep learning–based PASE embeddings [27] for our audio files. PASE represents the information contained in a raw audio instance through a list of encoded vectors. To ensure that the encoded vectors contain the same information as the input audio file, it decodes various properties of the audio file, including the audio waveform, log power spectrum, MFCCs, four prosody features (interpolated logarithm of the fundamental frequency, voiced or unvoiced probability, zero-crossing rate, and energy), and local InfoMax, from the encoded vectors. The encoded vectors must retain relevant information about the input audio file to decode all these properties successfully.

As these properties represent the inherent characteristics of the input audio file rather than any task-specific features, they have been used to solve a host of downstream tasks, such as speech classification, speaker recognition, and emotion recognition. Therefore, we also used them for PD detection.

### Experiments

For each feature set, we applied a standard set of machine learning algorithms, such as SVM [40], XGBoost [41], LightGBM [42], and random forest [43], to classify PD versus non-PD. SVM separates the data into several classes while maintaining the maximum possible margin among the classes. A random forest is built as an ensemble of decision trees; each decision tree builds a tree using a subset of features and learns if-else type decision rules to make a prediction. We also used XGBoost and LightGBM, algorithms based on gradient boosting where they build successively better models by refining the models at hand.

We used a leave-one-out cross-validation training strategy; using this strategy, one sample of data from the data set is left out, and the other n−1 samples are used to create a model and predict the remaining sample. We used metrics such as binary accuracy and AUC to evaluate our model’s performance. AUC is the area under the receiver operating characteristics (ROC) curve. The ROC curve is constructed by calculating the AUC produced by taking the ratio of the true-positive rate and the false-positive rate while varying the decision threshold. AUC has the highest value of 1, which denotes that the two classes can be separated perfectly, whereas an AUC value of 0.5 indicates that the model cannot distinguish between the two classes. Because our data set was imbalanced, AUC is a much better metric for understanding the true performance of our model. To reduce the effects of data imbalance, we used data augmentation techniques such as the synthetic minority oversampling technique [44] and SVM synthetic minority oversampling technique [45].

### Model Interpretation Technique

To interpret the models, we used the Shapley additive explanations (SHAP) technique based on the Shapley value. The Shapley value is a game-theoretic concept of distributing the payouts fairly among players [46]. In the machine learning context, each individual feature of an instance can be thought of as a player, and the payout is the difference between an instance’s prediction and the average prediction. We choose SHAP for two reasons: (1) it is well suited for explaining the output of any machine learning model, and (2) it is the only feature attribution method that fulfills the mathematical definition of fairness.

To use SHAP to explain gradient boosting and tree-based models such as XGBoost, Lundberg et al [47] introduced methods to provide a polynomial time model to compute optimized explanations. Their method can generate local interpretations—how the features affect one particular prediction of a data instance—and then combine those local interpretations to make global interpretations about features present in the entire data set. By setting up a class of features to condition on, they traverse the tree in the following manner: if we are traversing on a node that was split based on a feature we are conditioning on, we simply follow the decision path; otherwise, the results from the left and right subtrees originating from the current node are computed recursively, and their results are added through a weighted summation strategy, thus computing the SHAP value for the feature in question.

## Results

### Overview

The data were preprocessed, and both standard acoustic features (eg, pitch, jitter, shimmer, and MFCC) and deep learning–based audio embedding features, representing an audio clip as a feature vector, were extracted; henceforth, we call these *standard features* and *embedding features*. In the rest of the *Results* section, we discuss the results from the models built on the entire data set, interpretations of the best model on the entire data set, and results and interpretation from specialized models on gender-stratified and age-trimmed data sets.

### Detecting PD From the Entire Data Set

Table 3 contains the AUC and accuracy scores of the four machine learning models trained on the *standard features* and *embedding features* separately. Applying XGBoost on the standard features showed the best performance of 0*.*75 AUC and 0*.*74 accuracy. We also noticed that models trained on *standard features* work better than those trained on *embedding features.*

**Table 3:** Performance on the entire data set. The performance of various machine learning algorithms using the standard and embedding features on a data set combining data from both home and laboratory environments^a^.

Algorithm	Standard features		Embedding features	
	AUC^b^	Accuracy	AUC	Accuracy
SVM^c^	0.751	0.735	0.738	0.692
Random forest	0.745	0.720	0.726	0.708
LightGBM	0.753	0.720	0.737	0.693
XGBoost	*0.750* ^d^	*0.741*	0.722	0.689

^a^Models using standard features perform better than the models using embedding features in terms of both binary accuracy and area under the curve. Although the performance of the models is almost similar in terms of area under the curve metric, XGBoost outperforms others by considering both the area under the curve and accuracy metrics simultaneously.

^b^AUC: area under the curve.

^c^SVM: support vector machine.

^d^Variable outperforms all others by taking both area under the curve and accuracy into account.

### Model Interpretation

**Figure 5:**
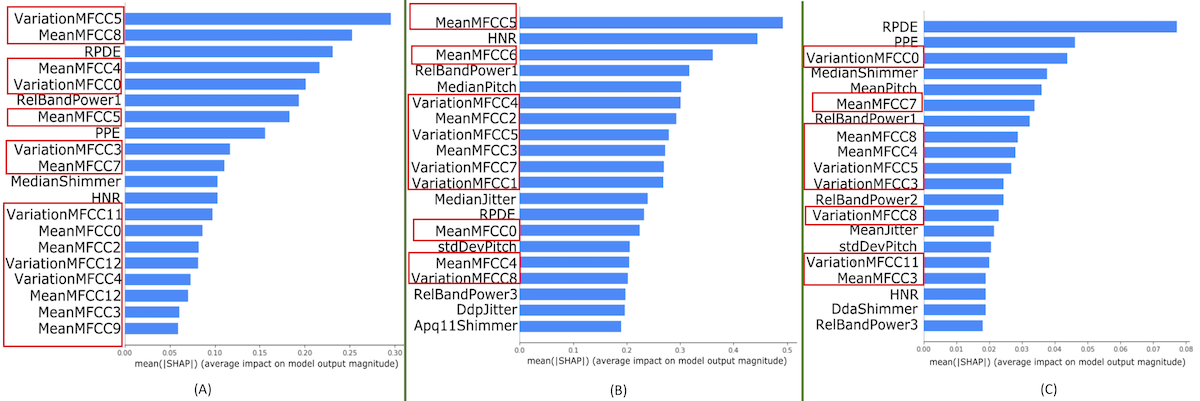
Shapley additive explanations analysis of our best performing models on 3 data sets: (A) main model (ie, entire data set), (B) female model (ie, female only), and (C) age-trimmed model (all subjects are older than 50 years).

The goal of SHAP is to explain the model’s prediction of any instance as a sum of contributions from its feature values; if a data instance can be thought of as X_i_=[f_1_, f_2_,...f_N_], SHAP will assign a number to each of these f_j_ features, denoting the impact of that feature—both the magnitude and direction—on the model’s prediction. Then, all these local explanations are aggregated to create a global interpretation for the entire data set. A global interpretation is presented in the first part of Figure 5; the top 10 most impactful features, ranked by having the most impact to the least, are presented. To calculate each feature’s impact, all of its SHAP values across all the data instances are gathered, and then the mean of their absolute values is calculated.

Features that have an impact on the model’s performance are typically the spectral features: the mean values or the variation of MFCC in each spectrum range. Apart from that, some other complex features such as recurrence period density entropy (RPDE; a measure of uncertainty in F0 estimation), PPE (a measure of inability to maintain a constant F0), and harmonic-to-noise ratio (HNR) also affected the interpretation presented in the first part of Figure 5.

### Gender- and Age-Stratified Analysis

**Figure 6:**
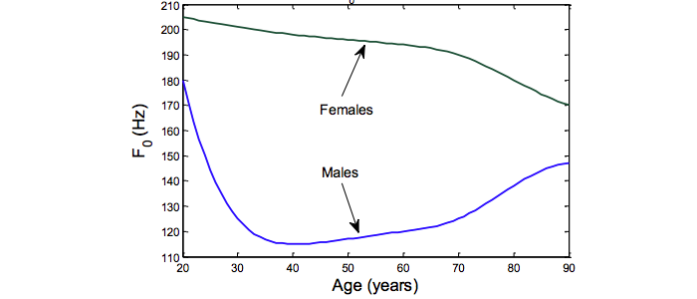
Changes in fundamental frequency F0 of voice as a function of gender and age (collected from Tsanas et al [13]).

The characteristics of a person’s voice are greatly influenced by age and gender. In Figure 6, we see that men and women display a changing characteristic in their voices as they get older. Female voices have a higher F0 value, but it decreases with age. Males typically have a higher F0 value in their youth, which decreases with age and then increases roughly after the age of 45 years. Therefore, it can produce confounding effects when analyzing PD from audio, where the machine learning model uses audio features to detect PD. To minimize the effect of confounding factors, researchers trained separate models on data from male and female participants [28] or analyzed an age-trimmed data set by considering data from participants older than 50 years [4,29].

### Building Specialized Models for Each Gender- and Age-Trimmed Analysis

The performance metrics of the machine learning models trained on the male, female, and age-trimmed data sets are shown in Table 4. By comparing the performance with metrics presented in Table 3, we can see that the models that used male- or age-trimmed data sets performed at par or better than the models that used the whole data set to train. However, there was a performance drop in the models using the female data set. Table 1 shows that females are overrepresented in the non-PD group and underrepresented in the PD group, leading to data imbalance and possibly lowering performance for the female-only model.

**Table 4:** Gender- and age-stratified models. Three separate data sets were constructed: a male data set with male participants, a female data set with female participants, and age-trimmed data set by excluding the participants younger than 50 years. For each of these data sets, a separate model was constructed, and its performance is reported below (N=726).

Algorithm	Male (n=415)		Female (n=477)			Age-trimmed (male, n=366 and female, n=426)	
	AUC^a^	Accuracy	AUC	Accuracy	AUC		Accuracy
SVM^b^	0.795	0.717	0.659	0.763	0.755		0.723
Random Forest	0.758	0.702	0.699	0.788	0.739		0.713
LightGBM	0.725	0.665	0.717	0.768	0.749		0.712
XGBoost	0.762	0.717	0.682	0.771	0.742		0.704

^a^AUC: area under the curve.

^b^SVM: support vector machine.

We also analyzed the features that drive the performance of these specialized models through SHAP analysis. The second part of Figure 5 shows the most salient features ranked by their SHAP value and the distribution of the impact of each feature on the model’s decision-making. The most important features are still dominated by the MFCC-related features or complex features such as the HNR, relative band power in different frequency ranges (RelBandPower1, RelBandPower3), RPDE (uncertainty in F0 estimation), perturbation in F0 (DdpJitter), or perturbation in amplitude (Apq11Shimmer). However, one noticeable fact is that three-pitch and jitter-related features—MedianPitch (median principal frequency, StdDevPitch (SD in principal frequency) and MedianJitter (median variation in F0)—also affected the model’s prediction, which was not noticed in the SHAP analysis run on the all-data-model.

Similarly, we interpreted the salient features of the age-trimmed data set in the third part of Figure 5. We noticed that the most salient features usually come from the MFCC feature groups, complex features (RPDE, PPE, and HNR), relative band power (RelBandPower1, RelBandPower2, and RelBandPower3), and pitch-related features. In addition, the pitch-related features also drove the prediction of the model.

## Discussion

### Limitations

In all of our experiments, we chose leave-one-out cross-validation to maintain uniform experimental settings between different models, data augmentation, and data set combinations because K-fold cross-validation can show high variance in performance based on how stratified the folds are. However, we acknowledge that our choice introduces the problem of overfitting and increases the computational complexity manifold. Therefore, we could not run extensive hyperparameter tuning to improve the performance of our models. By carefully stratifying the folds and maintaining the same fold settings for comparable experimental settings, K-fold cross-validation may enable us to achieve higher performance with lower computational complexity.

Moreover, our chosen metrics, accuracy and ROC AUC, can be overly optimistic because of the unbalanced nature of our data set. Metrics such as ROC–Precision-Recall balanced accuracy, and more information about sensitivity and specificity may shed more light on how we are performing in the minority class. In the future, we plan to apply other cross-validation techniques and better metrics to conduct our experiments and report our performance.

### Detecting PD From Regular Conversation

Some of the most common voice disorders induced by PD are dysphonia (distortion or abnormality of voice), dysarthria (problems with speech articulation), and hypophonia (reduced voice volume). Two speech-related diagnostic tasks are commonly used to detect PD by exploiting the changing vocal pattern caused by these disorders: (1) sustained phonation (the participant is supposed to utter a single vowel for a long time with constant pitch) and (2) running speech (the participant speaks a standard sentence). Little et al [12] developed features for detecting dysphonia in patients with PD. Tsanas et al [25] focused on the telemonitoring of self-administered sustained vowel phonation task to predict the Unified Parkinson’s Disease Rating Scale rating (on Rating Scales for Parkinson’s Disease 2003), a commonly used indicator for quantifying PD symptoms. These studies trained their models with data captured by sophisticated devices (eg, wearable devices, high-resolution video recorder, and the Intel at-home–testing-device telemonitoring system) that are often not accessible to all and are difficult to scale. The performance of these models can be significantly reduced when classifying data collected in home acoustics. In addition, correctly completing the sustained phonation task requires following a specific set of guidelines, such as completing the task in one breath, which can be difficult for older individuals.

In contrast, we analyzed the running speech task from the data collected by using a web-based data collection platform that can be accessed by anyone anywhere in the world and requires only an internet-connected device with an integrated camera and microphone. In addition, the running speech task does not require conforming to specific instructions and is more similar to regular conversation; therefore, the model can be potentially augmented to predict PD from a regular conversation—a potential game-changer in PD assessment. In the future, user-consented plug-ins could be developed for apps such as Alexa, Google Home, or Zoom, where audio is transmitted between persons. Anyone who consents to download the plug-in and uses it while on the phone, over Zoom, or giving virtual or in-person presentations could benefit from receiving an informal referral to see a neurologist, when appropriate. The plug-in would not store the participants’ data unless they opt to build a personalized profile to ensure privacy and ethical usage of our framework.

### Validating Model Interpretation

The features that SHAP found to have an impact on modeling decisions are well supported by previous research. For example, MFCC features have already proven to be useful in a wide range of audio tasks such as speaker recognition [48], music information retrieval [38], voice activity detection [49], and most importantly, in voice quality assessment [50]. Similarly, the high impact of the HNR and the measures of uncertainty in F0 estimation (RPDE) and inability to maintain a constant F0 (PPE) on the model’s output are in congruence with the findings from Little et al [12]. However, explaining the first part of Figure 5 in light of PD-induced vocal impairment is a difficult task. MFCC features are calculated by converting the audio signal into the frequency domain, and they denote how energy in the signal is distributed within various frequency ranges. Therefore, providing a physical interpretation of the SHAP values corresponding to the MFCC features is not straightforward. Similarly, Little et al [12] designed the RPDE and PPE features to model a sustained phonation task (uttering “ahh”) with the assumption that healthy participants will be able to maintain a smooth and regular voice pattern. In contrast, uttering multiple sentences introduces considerable variation in the data, adding a wide set of heterogeneous patterns. Therefore, the underlying assumptions behind constructing these features do not hold for our task of uttering multiple sentences.

We conduct an empirical validation of the SHAP output shown in the first part of Figure 5. We incrementally add one feature at a time to build a dynamic feature set, train successive models on that feature set, and report the accuracy and AUC performances. We see that the performance of our model saturates after adding 7 to 8 features. Therefore, we can say that the SHAP analysis teases out the most important features driving the model’s performance successfully.

### Why Not Stratified Analysis Only?

We built models inclusive of all genders for several reasons. First, there are potential shared characteristics among vocal patterns of all genders that can be relevant for detecting PD. Second, dividing the data set into two portions reduces the available training data for each model, which may, in turn, reduce the generalization capability of each model. In addition, our model analyzes data from patients of all ages. Although most people diagnosed with PD are older than 60 years, approximately 10% to 20% of the population diagnosed with PD are younger than 50 years, and approximately half of them are younger than 40 years [51]. As anecdotal evidence, Michael J Fox was diagnosed with PD at the age of 29 years [52], and Muhammad Ali had PD by 42 years [53]. In our data set, there were also a minority of patients with PD who were younger than 50 years (Figure 4). On the basis of these observations, we believe that our system should provide access to all people, irrespective of age. PD does not discriminate by age while affecting a person, and an automated system should not discriminate based on age and provide equitable services to people of all ages. However, these factors can function as confounders in PD analysis. Therefore, we provided additional analysis to ensure that our model does not use the idiosyncrasies of group-specific information to make predictions.

### Performance Excluding Laboratory Environment Data

When the data were collected in the laboratory, participants had access to a clinician providing support using a consistent recording setup and dedicated bandwidth. In contrast, the data collected in the home setting involved no assistance and included the real-world variability of heterogeneous recording setups and inconsistent internet speed. In theory, the data collected at the *laboratory* and *home* were very different from each other.

To ensure that our model works equally well without the *clean lab data*, we designed two experiments. In experiment 1, we removed clean lab data, which was approximately 7.4% (54/726) of the entire data set, retrained our model on the remaining 672 participants using the leave-one-out validation procedure, and calculated the performance metrics. In experiment 2, we randomly removed 7% (roughly 54 data points) of *home* data from the entire data set (while keeping the *lab* data intact), building a model with the remaining 93% data with the leave-one-out cross-validation method. Then, we conducted experiment 2 10 times and calculated the *average* performance from these 10 runs. We found that the AUC metric across these three experiments was fairly consistent, with a very small 0.015 decrease in AUC when removing lab data, demonstrating that our framework performs equally well across the *lab* and *home* data.

### Label Inconsistency and Predicting Tremor Score

Using the PARK [29] protocol, we collected one of the largest data sets of participants conducting a series of motor, facial expression, and speech tasks following the MDS-UPDRS PD assessment protocol [3]. Although we analyzed only the speech task in this study, the data set can be potentially used to automate the assessment of a large set of MDS-UPDRS tasks and facilitate early-stage PD detection, thus improving the quality of life for millions of people worldwide. However, deploying the data collection protocol on the web and facilitating access to anyone anywhere around the world comes at a cost. To date, all of our participants with PD have been clinically verified to be diagnosed with PD. Therefore, the labels of the PD data points are reliable. However, the participants without PD did not undergo clinical verification. Our data collection protocol asked them appropriate questions to check whether they had been diagnosed with PD and collected data when they answered in the negative. However, we cannot discount the possibility that a small subset of our population without PD is in the very early stage of PD and is oblivious about it. At present, there are estimated to be around 1 million patients with PD in the United States, out of a population of 330 million [51], yielding a PD prevalence rate of 0.3%. However, as our non-PD data set was largely tilted toward people older than 50 years, the rate in our data set could be higher than 0.3%. Even if we consider a liberal 1% prevalence rate, the number of individuals with undiagnosed PD in our control population is likely low (at most 4.6 persons). Therefore, we believe that the non-PD data labels are generally reliable. In the future, we plan to model tremor score in (0-4 range for each task; 0 for no tremor, and 4 for severe tremor) instead of a binary label following the MDS-UPDRS protocol to address this problem more thoroughly.

### Building a More Representative Data Set

The PARK protocol is web enabled, allowing anyone with access to the internet to contribute data. We plan to augment our data set by adding more non–native English speakers, females, and participants with PD. As our PD data are collected through contacts from local PD clinics and non-PD data through Amazon Mechanical Turk, most of our participants were from the United States or other English-speaking countries. To make our model more robust on data from non–native English speakers, we are in process of collecting both PD and non-PD data from non–native English-speaking countries. In addition, our current protocol can collect data from people with computer and internet access only, and therefore, it can potentially exclude underserved people. In the future, we plan to build desktop and mobile apps that can collect data offline to build a more inclusive framework.

Our best model for female data performed worse than its male counterparts, as shown in Table 4. We attribute this degraded performance PD or non-PD imbalance for female participants in our data set: the PD-to-non-PD ratio for females was 101 out of 300 (Table 1). Previous epidemiological studies have shown that both the incidence and prevalence of PD are 1.5 to 2 times higher in men than in women [54,55]. Therefore, any randomly sampled data set for PD will have a higher prevalence of males, contributing to models that are more biased toward males. Our immediate plan is to prioritize collecting balanced data from all genders, ages, and races across geographical boundaries, leading to a balanced data set.

Our data set also has the ubiquitous problem of data imbalance in diagnostic tests: the amount of data from participants without PD is 1.8 times more than their PD counterparts. Therefore, there is a risk that the model will be biased toward predicting the majority non-PD class as default and yield a high false-negative score. To address this, we plan to recruit more participants with PD in the future to make our data set more balanced. Another potential approach is to run the analysis on a subset of the data set produced by conducting proper age, gender, and matching of patients with and without PD.

As shown in Table 1, our participants with PD were diagnosed 7*.*88 (SD 5*.*41) years ago. Owing to limited data availability, we did not conduct an analysis on early-stage PD prediction. In the future, we plan to include more participants in the early stages of PD and build models that can detect them. We will especially focus on recruiting participants with PD in the age range of 20 to 40 years to analyze the very early-stage onset of PD among young adults. Furthermore, we plan to collect and analyze multiple speech instances from each person to reduce person-specific variability and obtain a more holistic view of their PD state.

### Increasing Model Performance

Although we consider AUC to be a better metric for our data set, our model performs 10% better than always choosing non-PD as the prediction in terms of binary accuracy. To be practically deployable in clinical settings, the performance needs to be improved further. We will focus on four promising avenues: making the data set balanced, designing better features capable of modeling the nuanced pattern in our data, making our model resilient to noise present in our data, and deconfounding the PD prediction from age and gender variables.

To remove noise, we plan to augment the techniques proposed in Poorjam et al [56] to automatically enhance our data quality by detecting the segments of data that conform to our experimental design. Moreover, as discussed above, gender and age can appear as confounding variables in the PD prediction task. In this paper, we have shown that our unified model and stratified sex-specific models have similar performance. However, we plan to build better models to systematically deconfound the effects of both age and gender variables while benefiting from them simultaneously. We can achieve this by incorporating the causal bootstrapping technique—a resampling method that considers the causal relationship between variables and negates the effect of spurious, indirect interactions, as outlined by Little and Badawy [57].

## Appendix

Multimedia Appendix 1 Description of the features.

## Abbreviations

AUC: area under the curve
HNR: harmonic-to-noise ratio
MDS-UPDRS: Movement Disorder Society-Unified Parkinson’s Disease Rating Scale
MFCC: mel-frequency cepstral coefficient
P2FA: Penn Phonetics Lab Forced Aligner Toolkit
PARK: Parkinson’s Analysis with Remote Kinetic-tasks
PASE: problem agnostic speech encoder
PD: Parkinson disease
PPE: pitch period entropy
ROC: receiver operating characteristics
RPDE: recurrence period density entropy
SHAP: Shapley additive explanations
SVM: support vector machine
