# Supplementary material for: Detecting Parkinson Disease Using a Web-Based Speech Task: Observational Study
Source: J Med Internet Res. 2021 Oct 19;23(10):e26305. doi: 10.2196/26305 (PMC8564663; doi:10.2196/26305)
Supplement: Multimedia Appendix 1 [file jmir_v23i10e26305_app1.docx]

## Multimedia Appendix

### Description of Acoustic Features:

**Pitch Related Features:** Pitch denotes the rate of vibrations present in a sound. *MedianPitch* and *MeanPitch* denote the me- dian and mean fundamental frequency or pitch of the audio signal. *StdDevPitch* denotes the standard deviation of fundamental frequency f0.

**Jitter related features:** Jitter defines how much a signal deviates from its presumed true periodicity; it is often an undesired quantity if our signals are assumed to be periodic.

*MeanJitter* is the measure of jitter collected by calculating the mean variation of f0. *MedianJitter* is the jitter measure calculated using the median variation of f0. *LocalJitter* denotes the average of the absolute differences between consecutive period of a signal – divided by the average period. *RapJitter* – Relative Average Perturbation– is computed by the average absolute difference between a period and the average of that period and the two neighbouring periods; divided by the average period. *Ppq5Jitter* denotes the five-point Period Perturbation Quotient: the average absolute difference between a period and the average of it and its four closest neighbours – divided by the average period. *DdpJitter* denotes the average absolute difference between consecutive differences between consecutive periods, divided by the average period.

**Shimmer Related Features:** Shimmer is a measurement of amplitude instability in an audio signal; a normal voice will have minimal instability during sustained verbal phonation production. *MeanShimmer* is the Shimmer value by quantifying the mean variation of amplitude in voice signals. *MedianShimmer* is the Shimmer calculated using the median variation of amplitude. *LocalShimmer* calculates the average absolute difference between the amplitude of the consecutive periods in a signal divided by the average amplitude. *LocalDBShimmer* is the average of the absolute value of 10-based logarithm of the difference between the amplitudes of consecutive periods in the signal, multiplied by 20. *Apq3Shimmer* is the three-point Amplitude Perturbation Quotient: the average absolute difference between the amplitude of a period and the average of the amplitudes of its two neighbours – left and right – divided by the average amplitude. *Apq5Shimmer* and *Apq11Shimmer* are similar to *Apq3Shimmer*, but uses data from four and 10 neighbours respectively instead of two. *DdpShimmer* is three times the value of *Apq3Shimmer*

**MFCC:** Mel Frequency Cepstral Coefficients (MFCC)^50^ are used to understand the rate of energy changes in different spectrum bands of the speech: If a cepstral coefficient has negative value, it indicates that majority of spectral energy in that spectrum band is concentrated in the high frequencies; if a cepstral has positive value, it indicates that majority of spectral energy is concentrated in low frequencies. As we get several entries for each of the 13 spectral regions of MFCC, we take the mean (**MeanMFCC-[0-12]**) and mean variation (**VariationMFCC[0-12]**) for each of these spectral regions.

**Relative Band Power:** Relative band power features were calculated by checking how much power is present in four different spectrum of frequency windows in the range [0,500,1000,2000,4000] Hz. The power contained in these four regions are denoted by **RelBandPower[0-3]**. Through applying FFT, we convert the audio signal into frequency domain. Then, we calculate the power contained in the frequencies belonging to each bucket, aggregate them and calculate the median in each of these buckets

**Harmonic-to-noise(HNR) ratio** HNR denotes the ratio of desired signal and background noise; higher HNR indicates better quality of audio.

**Recurrence period density entropy (RPDE):** A perfectly recurrent time signal will maintain a strict time period. Recurrence period density entropy (RPDE) determines how much a signal is maintaining a strict periodicity after the signal is reconstructed in phase space ^26^. By aggregating the time-periods recorded in our signal, and calculating the entropy of those time-periods, we get a measure of how much variation is present in those time-periods. A perfectly healthy voice will be able to maintain sustained vibration, hence it should have an entropy close to zero. Finally, the RPDE values are normalized in the range [0,1] to be used as feature.

**Detrended fluctuation analysis (DFA):** As human voice is produced by turbulent air-flows through our vocal folds, degen- eration of voice-fold structure (due to age or diseases) can produce increased noise in speech^26^. Detrended fluctuation analysis (DFA) measures the extent of the stochastic self-similarity of the noise in the speech signal produced due to possible alteration in vocal fold structure. These kind of noises can be represented through a statistical scaling component on a range of physical scales; this scaling component is comparatively larger for people with voice disorders^14,26^.

**Pitch Period Entropy (PPE):** (Little et al. 2008) ^38^ introduces a new feature Pitch Period Entropy (PPE) to calculate the entropy present in the pitch of an audio signal. First, a standard time-signal of pitch is converted into the logarithmic domain to capture the logarithmic nature of speech generation and perception. Then, to remove the gender and person specific trends present in the pitch – as we know that females have higher pitch voices than males, and there exists individual differences in pitch – we apply a standard whitening filter. Then, we use calculate the probability density of the residual signal. For a healthy voice signal, most of the probabilities will be concentrated on a narrow range. However, the people with vocal disorders cannot maintain a sustained pitch for a long time, therefore, there probability distribution will be much more dispersed. This dispersion is calculated through entropy, which precisely calculates how much disorganization there is in a system. A lower entropy means that the pitch was sustained over a long time, a higher entropy indicates problems with the vocal cords and probably dysphonia as well.
